# No association between vitamin D deficiency and parathyroid hormone, bone density and bone turnover in a large cohort of HIV-infected men on tenofovir

**DOI:** 10.7448/IAS.17.4.19568

**Published:** 2014-11-02

**Authors:** Amanda Samarawickrama, Sophie Jose, Caroline Sabin, Karen Walker-Bone, Martin Fisher, Yvonne Gilleece

**Affiliations:** 1 Clinical Investigation and Research Unit, Brighton and Sussex Medical School, Brighton, UK; 2 Department of Infection and Population Health, University College London, London, UK; 3 MRC Lifecourse Epidemiology Unit, University of Southampton, Southampton, UK; 4 Department of HIV and GU Medicine, Brighton and Sussex University Hospitals, Brighton, UK

## Abstract

**Introduction:**

Combination antiretroviral therapy (cART) may affect vitamin D [25(OH)D], parathyroid hormone (PTH), bone mineral density (BMD) and bone turnover (BT). Reduced BMD and secondary hyperparathyroidism have been reported with tenofovir (TDF). We investigated the associations between TDF and bone markers, especially in 25(OH)D-deficient patients.

**Materials and Methods:**

In a single-centre longitudinal study investigating BMD in HIV-positive men, serum 25(OH)D, calcium, phosphate, PTH and alkaline phosphatase (ALP) were measured. Lumbar spine, non-dominant total hip and non-dominant femoral neck BMD (g/cm^2^) were measured using dual-energy X-ray absorptiometry. BT was assessed by serum type 1 procollagen (P1NP) and carboxy-terminal collagen cross-links (CTX). Mann–Whitney U tests compared serum markers and BT, and t-tests compared BMD according to TDF in all and 25(OH)D-deficient patients.

**Results:**

A total of 422 men were recruited: mean age 47 (SD 9.8) years, 94% white ethnicity, 93% MSM, diagnosed HIV positive for median 9.6 (IQR 5.0,15.5) years, median CD4 547 (IQR 411,696) cells/µL, HIV RNA <40 copies/mL in 87% (96% of those on cART). 25(OH)D (nmol/L) was normal (>75), insufficient (50–75), deficient (25–50) and severely deficient (<25) in 14%, 29%, 50% and 7%, respectively. Of 381 men on cART, 77% were currently on TDF. TDF was not associated with median calcium (p=0.69) or phosphate (p=0.52), but patients had higher (but normal) median ALP [81 (IQR 69,103) vs. 73 (IQR 60,89) IU/L, p=0.005) compared to non-TDF cART. There was no difference in the association between vitamin D and PTH according to whether someone currently was (r=0.11, p=0.06, Figure 1) or was not using TDF (r=0.12, p=0.29, Figure 1). TDF was also not associated with PTH, BMD or BT in either all patients on cART (Table 1a) or in patients with 25(OH)D deficiency (Table 1b).

**Conclusions:**

In this largely TDF-experienced cohort of HIV-positive men, there was no association between TDF and 25(OH)D deficiency, hyperparathyroidism, reduced BMD or increased BT, although patients on TDF had higher but normal ALP. We found no evidence to support additional monitoring of bone markers in patients on TDF regardless of 25(OH)D status.

**Figure 1:**
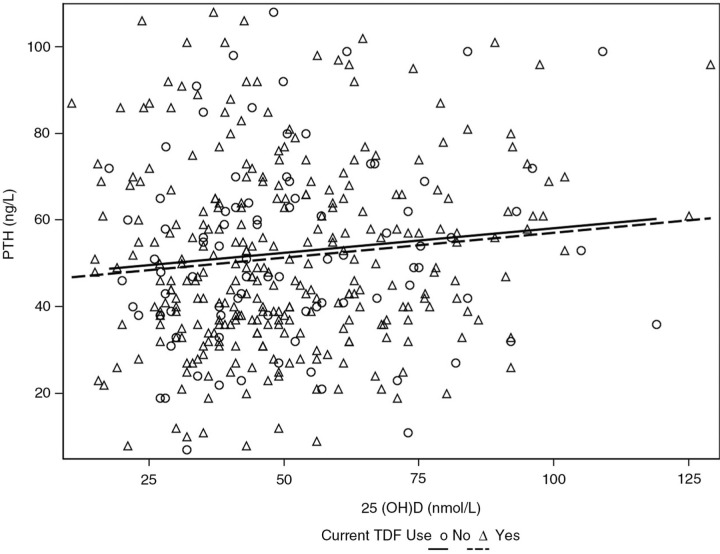
Association between 25(OH)D and PTH according to current TDF use.

**Table 1:** Bone markers, BMD, 25(OH)D and TDF use

		a) All patients			b) 25(OH)D<50 nmol/L	
						
	TDF cART (n = 293)	Non-TDF cART (n = 88)	P-value	TDF cART (n = 166)	Non-TDF cART (n = 49)	p Value
PTH, ng/L, median (IQR)	48 (36, 55)	51 (39, 65)	0.54	46 (36, 64)	47 (38, 62)	0.55
Lumbar spine BMD, g/cm^2^, mean (SD)	1.131 (0.146)	1.149 (0.174)	0.41	1.130 (0.153)	1.156 (0.176)	0.32
Non-dominant total hip BMD, g/cm^2^, mean (SD)	0.995 (0.133)	0.986 (0.144)	0.61	0.989 (0.140)	0.997 (0.151)	0.76
Non-dominant femoral neck BMD, g/cm^2^, mean (SD)	0.944 (0.124)	0.945 (0.180)	0.96	0.940 (0.129)	0.935 (0.137)	0.83
P1NP, ng/mL, median (IQR)	13.61 (5.55, 32.37)	10.95 (5.52, 24.83)	0.52	14.49 (5.83, 40.68)	12.66 (6.48, 23.95)	0.60
CTX, ng/mL, median (IQR)	1.94 (0.85, 4.84)	2.39 (1.02, 5.33)	0.29	2.02 (1.03, 4.84)	2.48 (1.17, 5.65)	0.32

